# LMTK2 binds to kinesin light chains to mediate anterograde axonal transport of cdk5/p35 and LMTK2 levels are reduced in Alzheimer’s disease brains

**DOI:** 10.1186/s40478-019-0715-5

**Published:** 2019-05-08

**Authors:** Gábor M. Mórotz, Elizabeth B. Glennon, Patricia Gomez-Suaga, Dawn H. W. Lau, Eleanor D. Robinson, Éva Sedlák, Alessio Vagnoni, Wendy Noble, Christopher C. J. Miller

**Affiliations:** Department of Basic and Clinical Neuroscience, Maurice Wohl Clinical Neuroscience Institute, Institute of Psychiatry, Psychology and Neuroscience, King’s College London, 125 Coldharbour Lane Camberwell, London, SE5 9RX UK

**Keywords:** Lemur tyrosine kinase-2, Kinesin-1, Cyclin dependent kinase-5/p35, Axonal transport, Alzheimer’s disease

## Abstract

Cyclin dependent kinase-5 (cdk5)/p35 is a neuronal kinase that regulates key axonal and synaptic functions but the mechanisms by which it is transported to these locations are unknown. Lemur tyrosine kinase-2 (LMTK2) is a binding partner for p35 and here we show that LMTK2 also interacts with kinesin-1 light chains (KLC1/2). Binding to KLC1/2 involves a C-terminal tryptophan/aspartate (WD) motif in LMTK2 and the tetratricopeptide repeat (TPR) domains in KLC1/2, and this interaction facilitates axonal transport of LMTK2. Thus, siRNA loss of KLC1 or mutation of the WD motif disrupts axonal transport of LMTK2. We also show that LMTK2 facilitates the formation of a complex containing KLC1 and p35 and that siRNA loss of LMTK2 disrupts axonal transport of both p35 and cdk5. Finally, we show that LMTK2 levels are reduced in Alzheimer’s disease brains. Damage to axonal transport and altered cdk5/p35 are pathogenic features of Alzheimer’s disease. Thus, LMTK2 binds to KLC1 to direct axonal transport of p35 and its loss may contribute to Alzheimer’s disease.

**Electronic supplementary material:**

The online version of this article (10.1186/s40478-019-0715-5) contains supplementary material, which is available to authorized users.

## Introduction

Dynamic changes to protein phosphorylation underpin many aspects of neuronal function. p35 is a binding partner and activator subunit of the neuronal serine-threonine kinase cdk5 [7, 23, 43, 47]. Together with cdk5, p35 regulates a variety of fundamental physiological processes within neurons including synaptic vesicle trafficking, dopaminergic and glutaminergic signalling, and axonal functions involving axonal outgrowth and the regulation of transport of cargoes through axons (axonal transport) [1, 7, 20, 36, 43]. In addition, damage to p35 leading to altered cdk5/p35 activity is believed to contribute to the pathogenesis of Alzheimer’s disease [7, 41, 43]. Despite the major roles that p35 plays within axons and synapses, the mechanisms by which it is transported into and through axons to these locations are unknown. Understanding these mechanisms is essential for properly comprehending p35 and cdk5 function in neurons and also its dysfunction in Alzheimer’s disease.

In addition to binding cdk5, p35 also binds to LMTK2 (also known as brain-enriched kinase, apoptosis-associated tyrosine kinase-2, kinase/phosphatase/inhibitor-2, cdk5/p35 regulated kinase, lemur tail kinase-2 and KIAA1079) [18, 28]. LMTK2 is a member of the lemur family of kinases which are a structurally unique group of kinases that are anchored in the membrane by a membrane-spanning region located at their extreme N-termini and which contain an N-terminally located kinase domain with a long C-terminal “tail” [3, 17, 18, 46, 53, 56]. As such, they have been named after lemurs, the long-tailed Madagascan primates. Topography studies have confirmed earlier predictions that LMTK2 is orientated so that its C-terminal tail projects into the cytosol [37]. Although originally predicted to be a dual-specificity serine-threonine/tyrosine kinase, several studies have since shown that LMTK2 only targets serine and threonine residues [17, 27, 28, 53, 54]. In neurons, LMTK2 along with p35 is enriched in cell bodies and the Golgi but is also present in axons and axon termini [18].

Kinesin-1 is a major microtubule-associated molecular motor that mediates transport of a wide variety of cargoes within neurons [13]. In axons, microtubule orientation is nearly uniform with the plus ends pointing toward the synapse and the minus ends facing the cell body. Since kinesin-1 unidirectionally moves toward microtubule plus ends, in axons it mostly drives anterograde transport toward the synapse. A proportion of functional kinesin-1 comprises a hetero-tetramer of two kinesin-1 motor proteins and two kinesin-1 light chains; KLC1 and KLC2 are the best characterized light chains [13, 31]. Kinesin-1 contains ATPase activity and uses the chemical energy of ATP to drive conformational changes that generate motile force; in contrast, the KLCs are mainly involved in binding of cargoes.

The importance of kinesin-1 driven axonal transport suggests that any disruption to this process may be detrimental to neurons. Indeed, damage to axonal transport is strongly implicated in Alzheimer’s disease [2, 5, 9, 30, 55]. Here we show that LMTK2 interacts with KLC1/2 and that this interaction facilitates its anterograde transport through axons. We also show that LMTK2 mediates the formation of a complex containing KLC1 and p35 and that siRNA loss of LMTK2 disrupts axonal transport of both p35 and cdk5. Finally, we show that LMTK2 levels are reduced in affected regions in post mortem Alzheimer’s disease brains. Loss of LMTK2 may therefore contribute to Alzheimer’s disease by effects on axonal transport of p35 and cdk5.

## Materials and methods

### Plasmids and siRNAs

pCI-neo control empty vector was from Promega. Myc-tagged mouse LMTK2, FLAG-tagged mouse KLC1 and KLC2, hemagglutinin (HA)-tagged human cdk5 and HA-, EGFP- and DsRed-tagged p35 plasmids were as described previously [12, 27, 51, 58]. C-terminal EGFP-tagged LMTK2 was generated by PCR amplification of LMTK2 [27] so as to remove the stop codon and cloning into pEGFP-N1 (Clontech) as a *Xho*I-*Hind*III fragment. Primers were 5′-AAACTCGAGATGCCGGGGCCGCCGGCG-3′ and 5′-AAAAAGCTTCTAGTCCTTGTCTCCGTCTTCAC-3′. N-terminally myc-tagged mouse kinesin-1A was generated by PCR from kinesin-1A IMAGE clone (BC058396; GeneService) and cloning into pCI-neo as a *Nhe*I-*Nhe*I fragment. Primers were 5′-AAAGCTAGCGAACAAAAACTCATCTCAGAAGAGGATCTGATGGCGGAGACTAACAACGAA -3′ and 5′-AAAGCTAGCTTAGCTGGCTGCTGTCTCTT-3′. N-terminal EGFP-tagged cdk5 was generated by subcloning HA-cdk5 into pEGFP-C1 (Clontech) as a *Bam*HI-*Bam*HI fragment. Mutant proteins were generated using a QuikChange II XL site-directed mutagenesis kit (Agilent Technologies) according to the manufacturer’s instructions with the following primer pairs:

LMTK2(WE477/478AA) 5′-AGCTTTGAGTATGTGGCGGCGGCTGCCAAGCACGAC-3′ and 5′-GTCGTGCTTGGCAGCCGCCGCCACATACTCAAAGC-3′.

LMTK2(WD1388/1389AA) 5′-GGCGGAGGCTTTGAAGCGGCCGATGACTTCTCCCCG-3′ and 5′-CGGGGAGAAGTCATCGGCCGCTTCAAAGCCTCCGCC-3′.

KLC1(N302L) 5′-GGCAGCGACTCTGAACCTCCTAGCAGTACTGTACGG-3′ and 5′-CCGTACAGTACTGCTAGGAGGTTCAGAGTCGCTGCC-3′.

KLC2(N287L) 5′-GGCTGCGACCCTCAACCTTCTGGCTGTTCTCTACGGC-3′ and 5′-GCCGTAGAGAACAGCCAGAAGGTTGAGGGTCGCAGCC-3′.

All constructs were verified by sequencing.

LMTK2, KLC1 and control siRNAs (Accell range) were from Dharmacon Horizon Discovery. LMTK2 siRNA was a pool of four constructs: 5′-GCCUGAGCUUUGAGUACGU-3′, 5′-CCCUUGGGUUUGUUGAAUU-3′, 5′-GUGUGCAGUUUAAUGGUAC-3′ and 5′-GAAAUAGACUUUAAGGAAU-3′.

KLC1 siRNA was 5′-GGGUCGUCUUUUGGAGAGU-3′.

### Antibodies

The following primary antibodies were used in this study:

rabbit anti-cdk5 (C-8, Santa Cruz; 1/500 immunoblot (IB)), mouse anti-FLAG (M2, Sigma; 1/250 immunoprecipitation (IP), 1/2000 IB), rabbit anti-GFP (Abcam; 1/250 IP; 1/5000 IB), mouse anti-GFP (B-2, Santa Cruz; 1/1000 IB), rabbit anti-HA (Sigma; 1/2000 IB; 1/500 immunofluorescence (IF)), mouse anti-kinesin-1 (H2, Millipore; 1/3000 IB), rabbit anti-KLC1 (H-75, Santa Cruz; 1/2000 IB), goat anti-KLC1 (L-15, Santa Cruz; 1/100 PLA), rabbit anti-LMTK2 (ab24782 Abcam; 1/500 IB), rabbit anti-LMTK2 (EPR7836(2); Abcam; 1/500 IB), rabbit anti-LMTK2 (described previously [18] 1/100 PLA), mouse anti-myc (9B11, Cell Signaling; 1/250 IP; 1/2000 IB; 1/2000 IF), mouse anti neuron specific enolase (NSE; Dako; 1/50,000 IB), rabbit anti-p35 (C-19, Santa Cruz; 1/100 PLA), rabbit-anti p35/25 (C64B10; Cell Signaling; 1/2000 IB), mouse anti-tubulin (DM1A, Sigma; 1/20,000 IB), mouse anti-β-tubulin isotype III (SDL.3D10; Sigma; 1/500 IF).

### Cell culture and transfection

HEK293 cells were grown in Dulbecco’s modified Eagle’s medium with 4.5 g/l glucose (GE Healthcare) supplemented with 10% (*v*/*v*) fetal bovine serum and 2 mM L-glutamine. Cells were transfected using TurboFect (Thermo Scientific) or with polyethylenimine MAX (Polysciences) according to the manufacturer’s instructions. Cells were analysed 24 h post-transfection.

Rat cortical neurons were obtained from embryonic day 17 embryos, plated on poly-L-lysine coated 18 mm diameter glass coverslips (Marienfield GmbH & Co.KG) or on 35 mm diameter glass bottom μ-dishes (Ibidi GmbH) and cultured in Neurobasal medium containing B27 supplement (Invitrogen), 2 mM L-glutamine, 100 IU/ml penicillin and 100 μg/ml streptomycin. Neurons were transfected using Lipofectamine 2000 (Invitrogen) (2 μl/μg DNA in Opti-MEM) according to the manufacturer’s instructions and analysed 24 h post transfection on DIV6 or 7. siRNAs were applied at DIV2 for 96 h.

### SDS-PAGE and immunoblotting

Cells were harvested for SDS-PAGE and immunoblotting by scraping into SDS-PAGE sample buffer containing 2% (*w/v*) SDS, 100 mM dithiothreitol, 10% (*w/v*) glycerol, 0.1% (*w/v*) bromophenol blue and protease inhibitors (Complete Roche) in 50 mM Tris-HCl pH 6.8 and heating to 96 °C for 5 min. Other samples were prepared by addition of SDS-PAGE sample buffer and heating to 100 °C for 5 min. Samples from cell cultures were separated on 8 or 12% gels using Mini-PROTEAN 3 gel electrophoresis systems (Bio-Rad) with a discontinuous buffer system. Human post-mortem brain homogenates were separated on Novex 4–12% Tris-Glycine Plus Midi Protein gels (Invitrogen) using the XCell4 Sureloc Midi-Cell system (Invitrogen). Separated proteins were transferred to BioTrace NT nitrocellulose membrane (0.2 μm pore size; Pall Corporation) using a Mini Trans-Blot electrophoretic transfer cell (Bio-Rad) for 16 h or a Midi Trans-Blot electrophoretic transfer cell (Bio-Rad) for 2 h. Membranes were blocked with Tris-HCl buffered saline (TBS) containing 5% (*w*/*v*) milk powder and 0.1% (*w*/*v*) Tween-20 for 1 h or with Odyssey TBS blocking buffer (Li-Cor Biosciences) for 1 h. Membranes were probed with primary antibodies in blocking buffers supplemented with 0.1% (*w/v*) Tween-20 (TBS/Tween-20), washed in TBS/Tween-20 and incubated with horseradish peroxidase (HRP)-conjugated secondary antibodies in wash buffer, and developed using an enhanced chemiluminescence development reagent (GE Healthcare) and exposure on Hyperfilm ECL. Alternatively, blots were incubated with IRDye-conjugated secondary antibodies in wash buffer and proteins visualised using an Odyssey CLx near infrared imaging system (Li-Cor Biosciences).

Signals on Hyperfilm ECL developed immunoblots were quantified using ImageJ (version 1.50b; developed by Wayne Rasband, National Institute of Health, Bethesda, USA) after scanning with an Epson Precision V700 Photo scanner as described by us in earlier studies [34]; only signals within the linear optical density range were used for analyses. Signals obtained with Odyssey CLx imaging system were quantified by Image Studio Lite (version 5.2.5; Li-Cor Biosciences). LMTK2 signals obtained from human brain samples were normalised to NSE signals from the same membrane.

### Immunoprecipitation assays

Immunoprecipitation assays were performed essentially as described [34]. Briefly, cells were washed with phosphate buffered saline (PBS) and harvested by scraping into ice-cold immunoprecipitation lysis buffer (50 mM Tris-citrate pH 7.4; 150 mM NaCl; 1% (*v/v*) Triton X-100; 5 mM EGTA; 5 mM EDTA and protease inhibitors (Complete, Roche)) for 1 h. Following centrifugation at 15,000 x g for 30 min at 4 °C, samples were incubated with primary antibody for 16 h at 4 °C. Antibodies were captured using Protein G-sepharose beads (50% (*v/v*) in PBS supplemented with 0.1% (*v/v*) Triton X-100) for 2 h at 4 °C, washed in capture buffer and bound proteins eluted and prepared for SDS-PAGE by incubation in 50 μl SDS-PAGE sample buffer and heating at 96 °C for 5 min.

### Preparation of human brain samples for SDS-PAGE and immunoblotting

Post-mortem human frontal cortex and cerebellum samples from control and pathologically confirmed cases of Alzheimer’s disease were obtained from the Medical Research Council Neurodegenerative Diseases Brain Bank, King’s College London. All tissue collection and processing were carried out under the regulations and licensing of the Human Tissue Authority, and in accordance with the Human Tissue Act, 2004. Post mortem studies from some control, clinically non-demented individuals revealed early Braak stage pathologies. Frozen human brain tissues were prepared as 20% homogenates in ice-cold radioimmunoprecipitation assay (RIPA) buffer (50 mM Tris-HCl pH 7.4; 150 mM NaCl; 1 mM EDTA; 1% (*v/v*) Triton X-100; 0.5% (*w*/*v*) sodium deoxycholate; 0.1% (*w/v*) SDS) with protease and phosphatase inhibitor cocktails (Roche) using a Bio-Gen PRO200 rotor-stator homogeniser (Pro Scientific) for 20 s. Following homogenisation, each sample was sonicated three times for 3 s before being centrifuged at 13,000 x g for 20 min at 4 °C. Supernatants were collected and protein concentrations determined using a bicinchoninic acid protein concentration assay kit (Pierce) according to the manufacturer’s instructions. Protein concentrations were adjusted to the same concentration in each sample by adding RIPA and SDS-PAGE sample buffers. Samples were stored at − 80 °C and heated to 96 °C for 10 min prior to SDS-PAGE.

### Immunofluorescence microscopy

Cells were fixed in 4% (*w*/*v*) paraformaldehyde in PBS for 15 min, washed three times in PBS, quenched with 50 mM NH_4_Cl in PBS for 15 min and permeabilised with 0.2% (*v/v*) Triton X-100 in PBS for 3 min. Cells were blocked with 3% (*w/v*) bovine serum albumin in PBS for 30 min and incubated with primary antibodies in blocking solution for 1 h. Following washing with PBS, the primary antibodies were detected using donkey anti-mouse Igs coupled with Cy5 (Jackson Immuno Research), goat anti-mouse and anti-rabbit Igs coupled with AlexaFluor 488, or 546 (Invitrogen) in blocking solution. After washing in PBS the samples were mounted in fluorescence mounting medium (Dako) or Mowiol-DABCO containing 10% (*w*/*v*) Mowiol 4–88 (Calbiochem), 25% (*w/v*) glycerol and 1% (*w/v*) DABCO (1,4-diazobicyclo [2.2.2] octane) in 100 mM Tris–HCl pH 8.5.

Images were captured using a Leica DM5000 B microscope equipped with 40x/0.75NA HCX-PL-FLUOTAR objective, DFC360 FX camera and appropriate filter sets (Leica). Axonal LMTK2, p35 and cdk5 levels were each acquired using the same microscope settings and quantified using ImageJ. To do so, background corrected non-saturated fluorescent axonal and cell body density signals were acquired and each axonal signal then normalised to its corresponding cell body signal.

### Proximity ligation assays

Proximity ligation assays (PLAs) were performed with rabbit anti-LMTK2 and goat anti-KLC1, and with rabbit anti-p35 and goat anti-KLC1 antibodies using Duolink PLA probes and orange detection reagent (Sigma) according the manufacturer’s protocol. Briefly, cells were fixed and permeabilised as described above for immunofluorescence staining, blocked with PLA blocking buffer for 30 min at 37 °C and incubated with primary antibodies for 1 h. Following washing, samples were incubated with PLA probes for 1 h at 37 °C and after further washing, probes were ligated for 30 min at 37 °C and amplified for 100 min at 37 °C. Following PLAs, cells were incubated with tubulin primary antibody in PBS at 4 °C for 16 h, washed with PBS and incubated with fluorescent conjugated secondary antibody for 1 h in PBS. Following further washing, samples were mounted as described above. Control reactions to demonstrate the specificity of the assays involved omission of primary antibodies. Signals were quantified using ImageJ particle analysis function as described [42].

### Time-lapse microscopy

Axonal transport of EGFP- and DsRed-tagged LMTK2, p35 and cdk5 in living neurons was monitored using a Nikon Eclipse Ti-E microscope equipped with Intenslight C-HGFI light source (Nikon), EGFP and DsRed filter sets (Chroma Technology), CFI Apo Lambda S 60x/1.40 objective (Nikon) and an Andor Neo scientific complementary metal-oxide-semiconductor camera (Andor Technology). Movements were recorded using NIS-Elements AR software (Nikon). Particles were imaged 24–36 h post-transfection in Ibidi μ-dishes or by mounting coverslips in a Ludin imaging chamber (Life Imaging Services) filled with external solution (145 mM NaCl, 2 mM KCl, 5 mM NaHCO_3_, 1 mM MgCl_2_, 2.5 mM CaCl_2_, 10 mM glucose in 10 mM HEPES pH 7.0). Temperature was maintained at 37 °C during imaging using a microscope incubation chamber (Solent Scientific). Movements were recorded at 1 s time-lapse intervals and 100 ms exposure times. Kymographs were created using the Straighten and Kymograph plugins of ImageJ. In line with our previous studies, we chose cells expressing low levels of transfected proteins (as judged by the fluorescent protein signal) for analyses so as to avoid any possible artefacts produced by high levels of expression [1, 50].

### Statistical analyses

Statistical analysis was performed using Excel (Microsoft Corporation), Prism software (version 7.02; GraphPad Software Inc.) and SPSS (version 24.0.0.1; IBM). Statistical significance was determined by t-test or analysis of variance (ANOVA) followed by post-hoc test as described in figure legends. Data are plotted as mean ± s.e.m. All experiments were repeated at least three times.

## Results

### LMTK2 binds to KLCs via a C-terminal WD motif

**Fig. 1:**
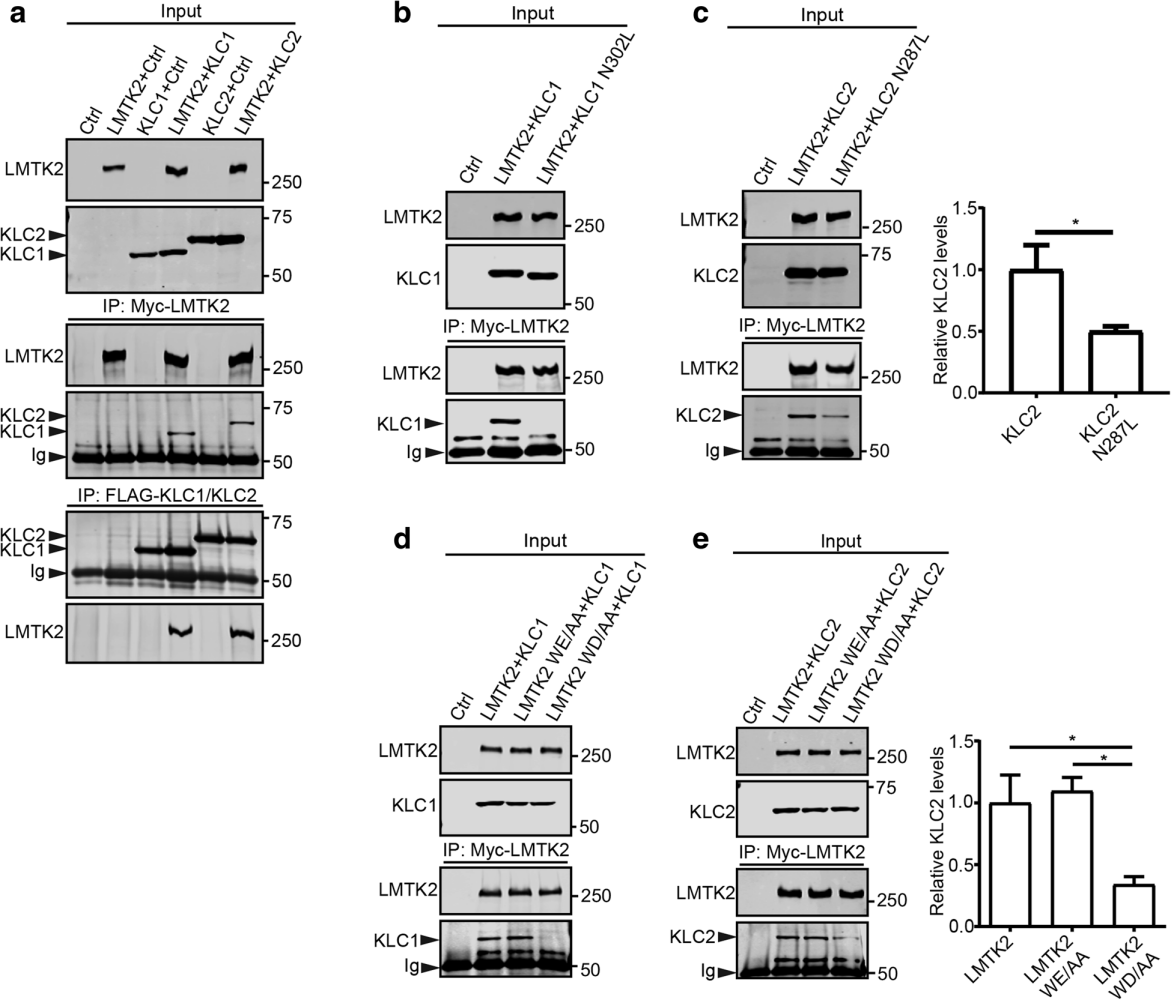
LMTK2 binds to both KLC1 and KLC2 via a C-terminal WD motif (a.a. 1388/1389). **a** LMTK2 binds to KLC1 and KLC2. HEK293 cells were transfected with either control empty vector (Ctrl), myc-LMTK2, FLAG-KLC1, FLAG-KLC2, myc-LMTK2 + FLAG-KLC1 or myc-LMTK2 + FLAG-KLC2. LMTK2, KLC1 and KLC2 were each immunoprecipitated via their epitope tags and bound KLC1/KLC2 or LMTK2 detected by immunoblotting. No signals for co-immunoprecipitated proteins were obtained in control cells transfected with only one plasmid which demonstrates the specificity of the assays. Both inputs and immunoprecipitates (IP) are shown. **b** and **c** Mutation of KLC1(N302) or KLC2(N287) to leucine within their TPR domains disrupts binding to LMTK2. HEK293 cells were transfected with control vector, myc-LMTK2 + FLAG-KLC1, myc-LMTK2 + FLAG-KLC1(N302L), myc-LMTK2 + FLAG-KLC2 or myc-LMTK2 + FLAG-KLC2(N287L). myc-LMTK2 was immunoprecipitated via the myc-tag and bound KLC1 (**b**) or KLC2 (**c**) detected on immunoblots. Mutant KLC1(N302L) did not bind to LMTK2 and KLC2(N287L) displayed reduced binding to LMTK2. Both inputs and immunoprecipitates are shown. Graph in (**c**) shows relative levels of KLC2 bound to LMTK2 in the immunoprecipitations following quantification of signals on immunoblots. Data were analysed by t-test. *N* = 5; error bars are s.e.m., *p < 0.05. Both inputs and immunoprecipitations (IP) are shown. **d** and **e** Mutation of LMTK2 WD (1388/1399) but not WE (477/478) to AA disrupts binding to KLC1 and KLC2. HEK293 cells were transfected with control vector, myc-LMTK2 + FLAG-KLC1/KLC2, myc-LMTK2(WE477/478) + FLAG-KLC1/KLC2 or myc-LMTK2(WD1388/1389) + KLC1/KLC2. LMTK2 was immunoprecipitated via its epitope tag and bound KLC1 (**d**) or KLC2 (**e**) detected by immunoblotting. Mutation of LMTK2 WD/AA (1388/1389) abolished binding of KLC1 and markedly inhibited binding of KLC2. Both inputs and immunoprecipitates are shown. Bar chart in (**e**) shows relative levels of KLC2 bound to LMTK2 in the immunoprecipitations following quantification of signals on immunoblots. Data were analysed by one-way ANOVA and Tukey’s post hoc test. *N* = 3; error bars are s.e.m., *p < 0.05.

We tested whether LMTK2 might bind to KLC1 and KLC2 in immunoprecipitation assays. To do so, we transfected HEK293 cells with either empty pCIneo control vector, myc-tagged LMTK2 (myc-LMTK2), FLAG-tagged KLC1 (FLAG-KLC1), FLAG-KLC2, myc-LMTK2 + FLAG-KLC1 or myc-LMTK2 + FLAG-KLC2 and monitored the LMTK2-KLC1/KLC2 interaction on immunoblots following immunoprecipitation of either LMTK2, KLC1 or KLC2 via their epitope tags. LMTK2 bound to both KLC1 and KLC2 in these assays and the interaction was only detected in LMTK2 + KLC1/KLC2 co-transfected cells which demonstrates the specificity of the assays (Fig. 1a).

The mechanisms by which cargoes attach to kinesin-1 motors are not fully understood but one route involves interactions between the TPR domains in KLC1/2 and cargo located tryptophan-aspartate/tryptophan-glutamate (WD/WE) motifs [10, 33, 40]. Mutation to leucine of conserved asparagines within the KLC TPRs (KLC1(N302) and KLC2(N287)) has been shown to disrupt WD/WE cargo binding [40, 59]. We therefore studied binding of LMTK2 to mutant KLC1(N302L) and KLC2(N287L) using immunoprecipitation assays similar to those described above. Mutant KLC1(N302 L) did not bind and KLC2(N287 L) displayed reduced binding to LMTK2 in these assays (Fig. 1b, c). Differences in binding of WD/WE containing proteins to KLC1 and KLC2 have been described previously [10, 49].

LMTK2 contains two conserved WE/WD motifs that might potentially interact with the KLC TPR domains (WE477/478 and WD1388/1389). We therefore mutated LMTK2(WE477/478) and LMTK2(WD1388/1389) separately to double alanine, and again monitored binding of the mutants to KLC1 and KLC2 in immunoprecipitation assays. Mutant LMTK2(WE477/478AA) bound to both KLC1 and KLC2 in a manner indistinguishable from wild-type LMTK2 (Fig. 1d, e). However, mutant LMTK2(WD1388/1389AA) did not bind to KLC1 and displayed reduced binding to KLC2 (Fig. 1d, e). These findings complement the above studies showing that mutation of the KLC1 TPR domain had a more potent effect on binding to LMTK2 than mutation of the KLC2 TPR domain (Fig. 1b, c). We therefore focussed on the LMTK2-KLC1 interaction. KLC1 has been shown to be involved in transporting a large number of cargoes in axons [11, 44, 52].

**Fig. 2:**
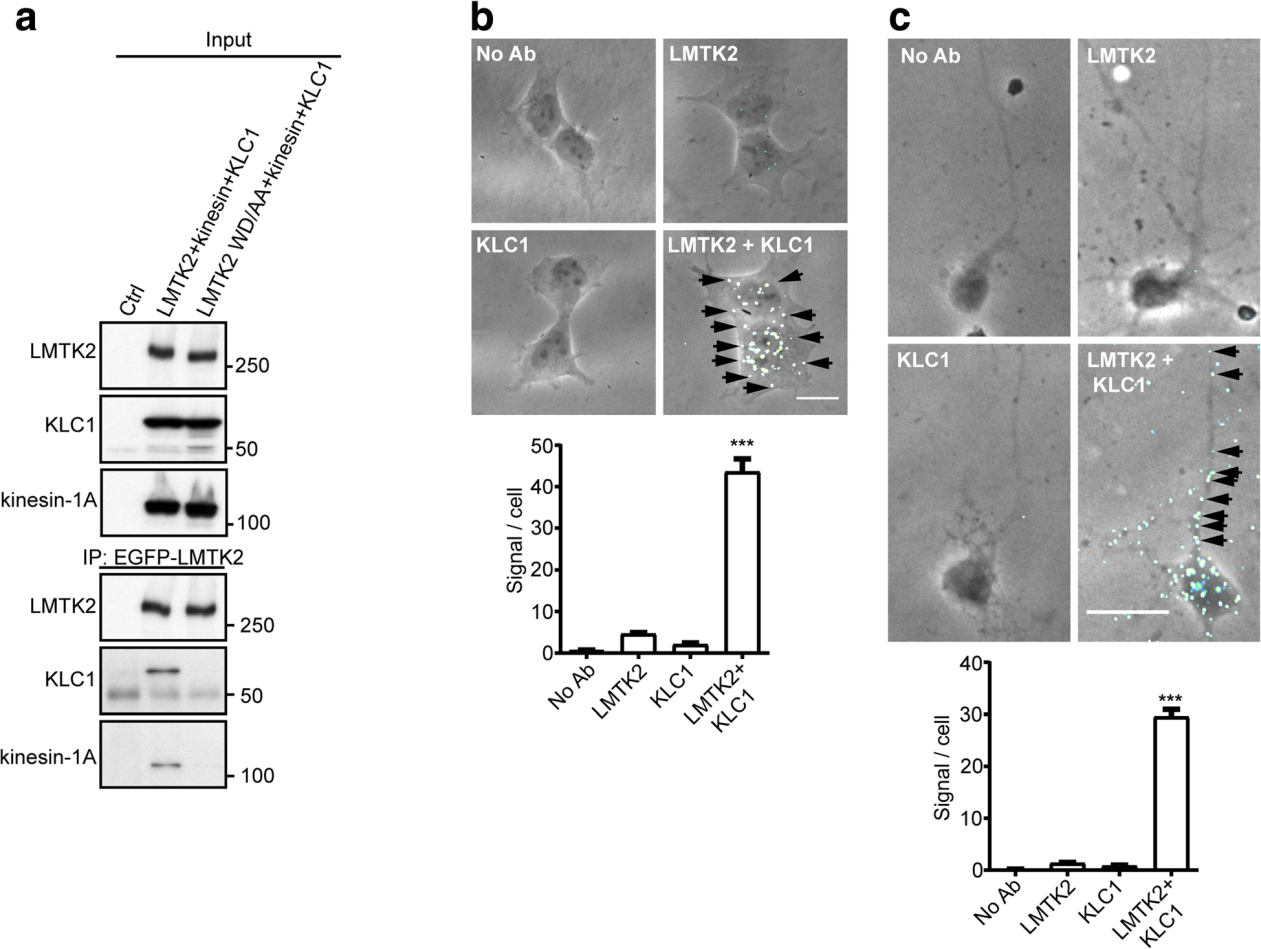
LMTK2 is complexed with both KLC1 and kinesin-1 and mutation of LMTK2(WD1388/1389) to AA disrupts this binding. **a** HEK293 cells were transfected with either control empty vector (Ctrl), EGFP-LMTK2 + FLAG-KLC1 + myc-kinesin-1A or EGFP-LMTK2(WD1388/1389AA) + FLAG-KLC1 + myc-kinesin-1A. LMTK2 was immunoprecipitated via the EGFP-tag and bound KLC1 and kinesin-1 detected by immunoblotting. Both inputs and immunoprecipitations (IP) are shown. **b** and **c** Endogenous LMTK2 and KLC1 are complexed in HEK293 cells (**b**) and rat cortical neurons (**c**). LMTK2-KLC1 PLAs were performed using rabbit anti-LMTK2 and goat anti-KLC1 antibodies. Control experiments involving omission of both or each primary antibody are also shown. Representative phase contrast images with PLA signals of cells are displayed; arrows indicate PLA signals and in (**c**) show signals in axons. To facilitate viewing of signals in these phase contrast images, the PLAs colours were electronically changed from red to pale blue. Scale bars = 20 μm. Graphs show PLA signals per cell in the different experiments. Data were analysed by Welch’s ANOVA and Games-Howell post hoc test. **b** *N* = 40–46 cells, **c** *N* = 43–54 cells; error bars are s.e.m., ***p < 0.001

We next used immunoprecipitation assays to determine whether LMTK2 is complexed with the kinesin-1 motor. To do so, we immunoprecipitated EGFP-tagged wild-type LMTK2 or LMTK2(WD1388/1389AA) from FLAG-KLC1 + myc-kinesin-1 co-transfected HEK293 cells. Both KLC1 and kinesin-1 bound to LMTK2 but not LMTK2(WD1388/1389AA) in these assays (Fig. 2a). Finally, we used PLAs to test whether endogenous LMTK2 and KLC1 were associated with each other in cultured HEK293 cells and rat cortical neurons. These PLAs produced positive signals which in the neurons included in cell bodies and axons (Fig. 2b, c). In both HEK293 cells and neurons, confocal analyses revealed that the KLC1-LMTK2 PLA signals were particularly abundant in perinuclear regions but not within nuclei. This is in agreement with previous studies which show that LMTK2 is enriched in these areas including the Golgi [18]. Thus, LMTK2 binds to both KLC1 and KLC2 via a conserved WD motif in its C-terminal tail domain (residues 1388/1389), this binding involves the KLC TPR domains and the interaction of LMTK2 with KLC1 facilitates its binding to the kinesin-1 motor complex.

### Axonal transport of LMTK2 involves its binding to KLC1

**Fig. 3:**
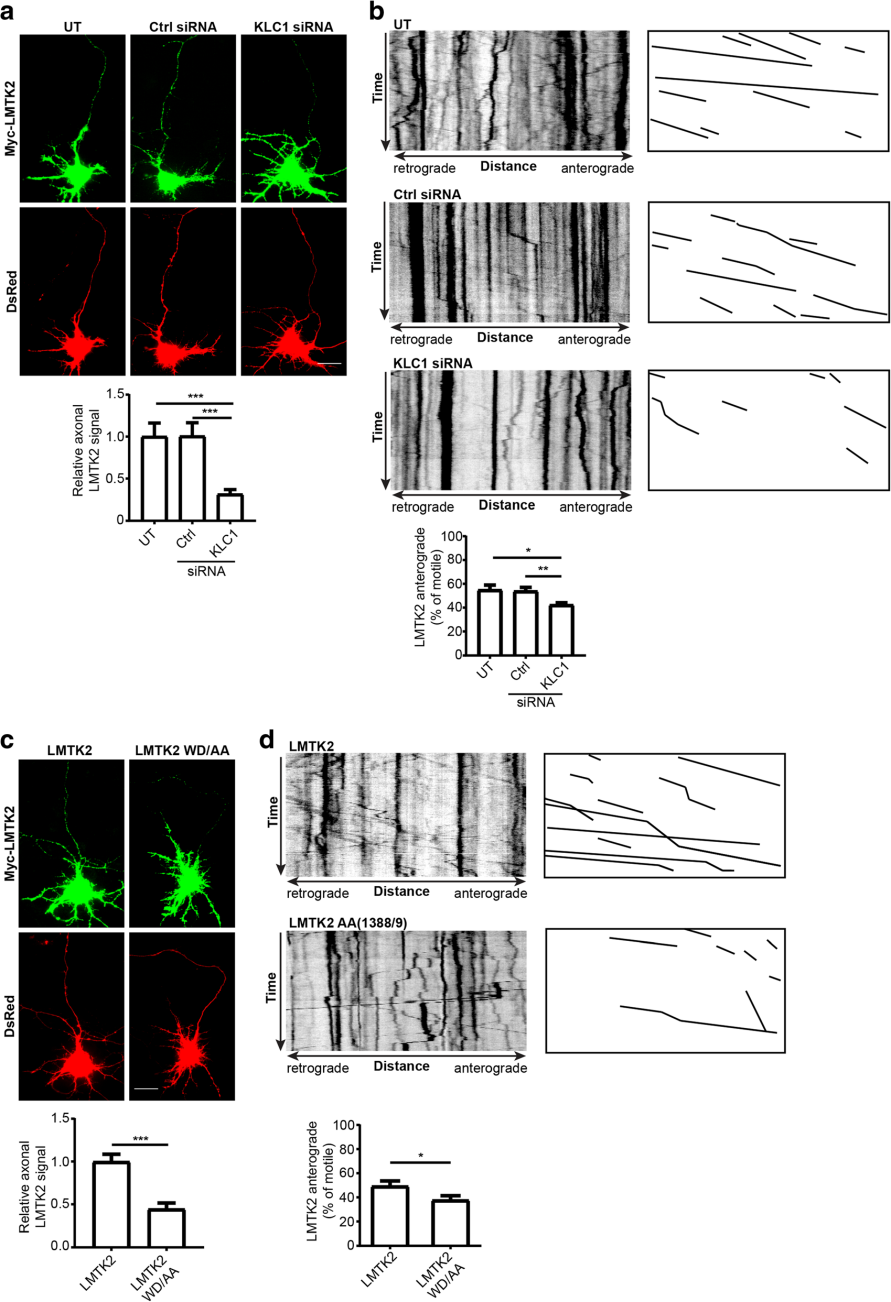
siRNA loss of KLC1 and mutation of LMTK2 WD (1388/1389) disrupt axonal transport of LMTK2. **a** and **b** siRNA loss of KLC1 disrupts axonal levels and anterograde axonal transport of LMTK2. Neurons were either untreated (UT) or treated with control or KLC1 siRNAs and then co-transfected with myc-LMTK2 and DsRed to reveal neuronal architecture in (**a**) or EGFP-LMTK2 alone in (**b**). **a** Neurons were fixed and transfected LMTK2 detected by immunostaining for its epitope tag. Representative images of neurons are displayed along with graph showing quantification of relative axonal LMTK2 levels. Scale bar = 20 μm. Data were analysed by Welch’s ANOVA and Games-Howell post hoc test; *N* = 28–43, error bars are s.e.m., ***p < 0.001. **b** Representative kymographs showing axonal transport of EGFP-LMTK2 in living neurons; for clarity anterogradely moving EGFP-LMTK2 are illustrated alongside, time = 180 s. Graph shows quantification of anterogradely moving LMTK2 in the different siRNA treated neurons. Data were analysed by Welch’s ANOVA and Games-Howell post hoc test; *N* = 15–23, error bars are s.e.m., *p < 0.05, **p < 0.01. **c** and **d** Mutation of LMTK2 WD (1388/1389) to AA disrupts axonal levels and anterograde axonal transport of LMTK2. **c** Neurons were co-transfected with DsRed and either myc-LMTK2 or myc-LMTK2(WD1388/1389AA) and LMTK2 detected in fixed cells by immunostaining for its epitope tag. Representative images of neurons are displayed along with graph showing quantification of relative axonal LMTK2 levels. Scale bar = 20 μm. Data were analysed by t-test; *N* = 28 and 31 neurons, error bars are s.e.m., ***p < 0.001. **d** Representative kymographs showing axonal transport of EGFP-LMTK2 and EGFP-LMTK2(WD1388/1389AA) in living neurons; for clarity, anterogradely moving EGFP-LMTK2 are illustrated alongside, time = 180 s. Graph shows quantification of anterogradely moving LMTK2 in the different transfected neurons. Data were analysed by t-test; *N* = 23–26, error bars are s.e.m., *p < 0.05

LMTK2 is present in neuronal cell bodies and axons [18]. To determine whether LMTK2 delivery into axons involves its binding to KLCs, we first studied how siRNA depletion of KLC1 affected the axonal distribution of transfected myc-LMTK2 in rat cortical neurons. KLC1 siRNAs have been characterised previously [51] and induced an approximate 80% reduction in protein levels without affecting expression of endogenous kinesin-1, LMTK2, p35 or cdk5 (Additional file 1: Figure S1a). Neuronal morphologies were determined by co-transfection of DsRed and LMTK2 was identified by immunostaining for its myc-tag. Compared to untreated or control siRNA treated neurons, KLC1 siRNA knockdown cells displayed a noticeable reduction in axonal LMTK2 labelling. To quantify this phenotype, we normalised axonal LMTK2 fluorescent signals to cell body signals in each neuron. These analyses revealed a significant reduction in axonal LMTK2 in the KLC1 siRNA knockdown cells (Fig. 3a).

To complement these KLC1 siRNA knockdown studies, we also monitored axonal localisation of transfected wild-type myc-LMTK2 and mutant myc-LMTK2(WD1388/1389AA) that does not bind to KLC1 in the cortical neurons. Compared to wild-type LMTK2, LMTK2(WD1388/1389AA) displayed reduced axonal labelling and we again quantified this phenotype by normalising axonal LMTK2 fluorescent signals to cell body signals in each neuron. These analyses confirmed that LMTK2(WD1388/1389AA) levels were significantly reduced in axons (Fig. 3c).

We next monitored how siRNA loss of KLC1 and mutation of LMTK2(WD1388/1389AA) affect axonal transport of transfected EGFP-LMTK2 in living rat cortical neurons using time-lapse microscopy. Movement of wild-type EGFP-LMTK2 was observed in both anterograde and retrograde directions and the mean anterograde velocity was 0.58 +/− 0.42 μm/s. This velocity for EGFP-LMTK2 movement is in line with the velocities described for other known kinesin-1/KLC1 cargoes such as calsyntenin-1, amyloid precursor protein (APP) and Kidins220/ARMS [4, 50, 51]. However, siRNA loss of KLC1 significantly reduced the numbers of anterogradely moving EGFP-LMTK2 cargoes (Fig. 3b). Likewise, compared to wild-type EGFP-LMTK2, the numbers of EGFP-LMTK2(WD1388/1389AA) anterogradely moving cargoes were significantly reduced (Fig. 3d). Thus, the binding of LMTK2 to KLC1 via its WD1388/1389 motif facilitates LMTK2 transport into and through axons.

We also calculated how siRNA loss of KLC1 and mutant LMTK2(WD1388/1389) affect the proportion of total moving LMTK2 cargoes (anterograde plus retrograde) and their anterograde velocities. Compared to control siRNA, KLC1 siRNA treatment did not significantly affect either of these parameters. However, LMTK2(WD1388/1389) showed reduced total movement (*p* < 0.001) compared to wild-type LMTK2 but this did not alter its velocity. These findings are in line with the LMTK2(WD1388/1389) mutant having a particularly potent effect on binding to KLC1.

### LMTK2 forms a complex with both KLC1 and p35

**Fig. 4:**
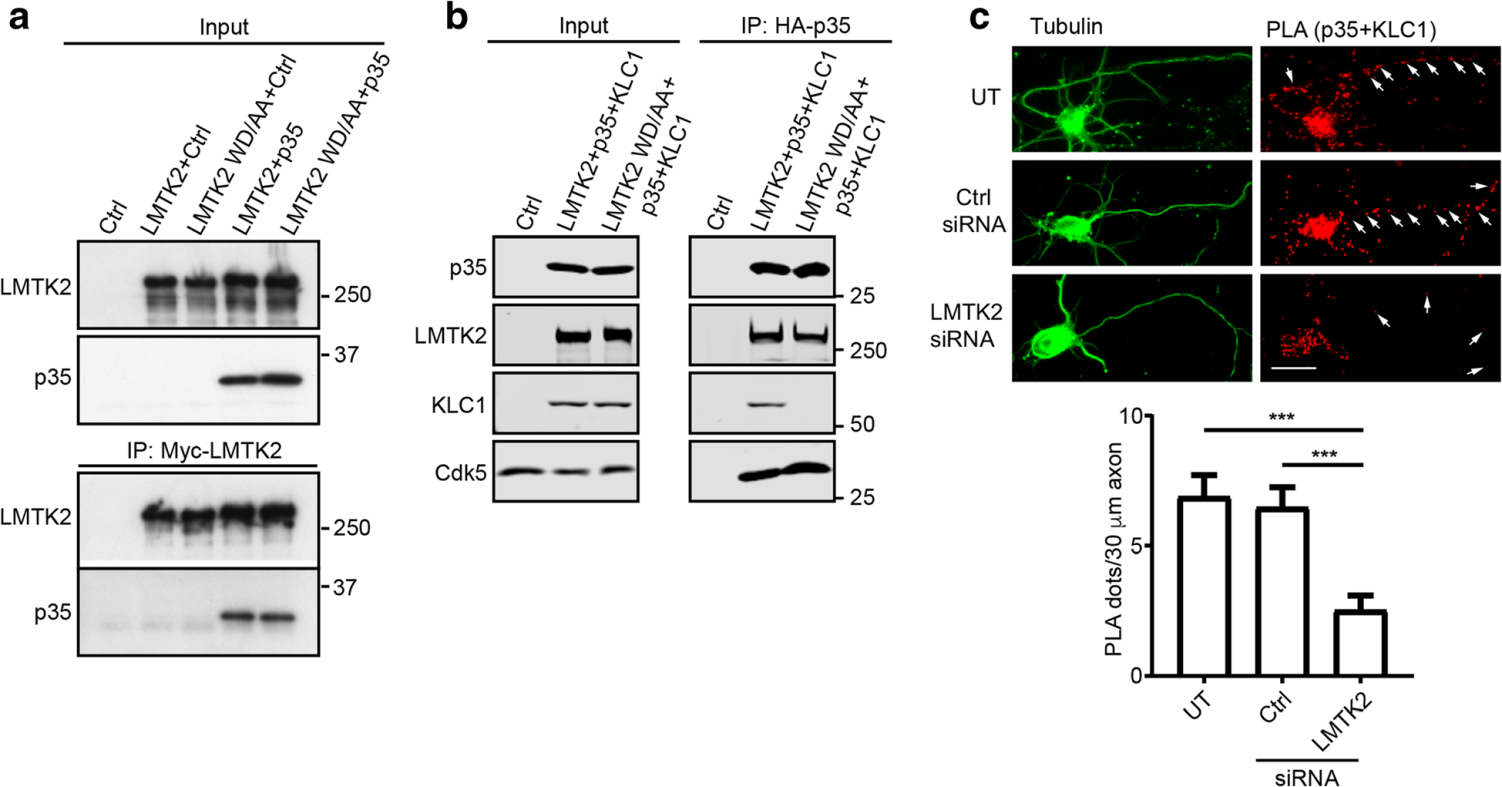
LMTK2, p35 and KLC1 form a complex. **a** Mutation of LMTK2(WD1388/1389AA) does not affect LMTK2 binding to p35. HEK293 cells were transfected with either control empty vector (Ctrl), myc-LMTK2, myc-LMTK2(WD/1388/1389AA), myc-LMTK2 + p35 or myc-LMTK2(WD1388/1389AA) + p35. LMTK2 was immunoprecipitated via the myc-tag and bound p35 detected by immunoblotting. Both inputs and immunoprecipitations (IP) are shown. **b** p35 forms a complex with cdk5, LMTK2 and KLC1, and mutation of LMTK2(WD1388/1389AA) specifically disrupts KLC1 binding to the complex. HEK293 cells were transfected with either control empty vector (Ctrl), HA-p35 + myc-LMTK2 + FLAG-KLC1 or HA-p35 + myc-LMTK2(WD1388/1389AA) + FLAG-KLC1. p35 was immunoprecipitated via the HA-tag and bound LMTK2, KLC1 and cdk5 detected on immunoblots. Both inputs and immunoprecipitations (IP) are shown. **c** LMTK2 mediates formation of a complex between KLC1 and p35 in cortical neurons. Neurons were either untreated or treated with control or LMTK2 siRNAs and PLAs for KLC1 and p35 performed using goat anti-KLC1 and rabbit anti-p35 antibodies. Neurons were also immunostained for tubulin to reveal architecture. KLC1-p35 signals are markedly reduced in both cell bodies and axons in LMTK2 siRNA treated neurons; arrows show PLA signals in axons. Graph shows quantification of PLA dots in axons in the different treated cells. Data were analysed by Welch’s ANOVA and Games-Howell post hoc test; *N* = 15–24, error bars are s.e.m., ***p < 0.001

LMTK2 also binds to p35 and this interaction involves amino acids within residues 391–632 of LMTK2 [18]. This region is located some distance from the KLC interaction motif (WD1388/1389) we identify above which suggests that LMTK2 may form a complex with both p35 and KLC1. To test this possibility, we first used immunoprecipitation assays to determine whether mutation of LMTK2(WD1388/1389) affected binding of p35 to LMTK2 in transfected HEK293 cells. As predicted, immunoprecipitated LMTK2 and LMTK2(WD1388/1389) bound equally well to p35 in these assays (Fig. 4a). We next tested whether LMTK2 formed a complex with KLC1 and p35 again using immunoprecipitation assays in transfected HEK293 cells. Like many other non-neuronal cells lines, HEK293 cells express endogenous cdk5 but not p35 [19]. HA-tagged p35 was immunoprecipitated from cells co-transfected with FLAG-KLC1 and either wild-type myc-LMTK2 or myc-LMTK2(WD1388/1389AA) that does not bind KLC1. p35 bound to endogenous cdk5 and both myc-LMTK2 and myc-LMTK2(WD1388/1389AA) in these assays. However, KLC1 was only detected in the p35 immunoprecipitates in the presence of wild-type LMTK2 (Fig. 4b). These findings demonstrate that the interaction between p35 and KLC1 is dependent upon LMTK2. We also used PLAs to detect interactions between endogenous p35 and KLC1 in untreated rat cortical neurons, or in neurons treated with control or LMTK2 siRNAs. LMTK2 siRNAs have been characterised previously [27] and led to an approximate 75% reduction of LMTK2 in the neurons (Additional file 1: Figure S1b). Control experiments involving omission of p35 or KLC1 antibodies demonstrated the specificity of these PLAs (Additional file 1: Figure S2). Robust PLA signals were detected between p35 and KLC1 in control cells but siRNA loss of LMTK2 induced a significant decrease in the number of these signals (Fig. 4c). Thus, LMTK2 mediates the formation of a complex containing both p35 and KLC1.

### LMTK2 is required for axonal transport of p35

**Fig. 5:**
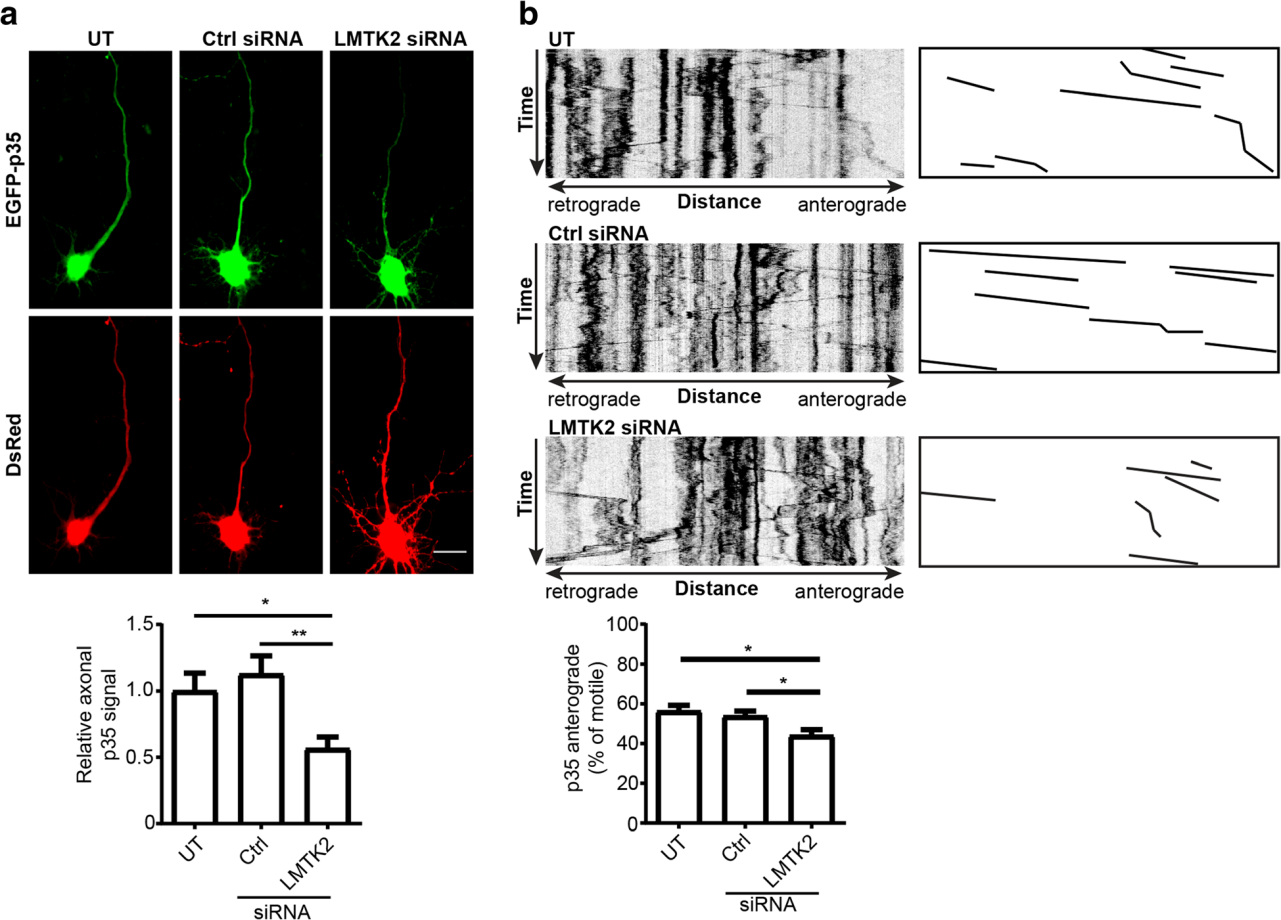
siRNA loss of LMTK2 reduces axonal levels and anterograde axonal transport of p35. **a** Neurons were either untreated (UT) or treated with control or LMTK2 siRNAs and then co-transfected with EGFP-p35 and DsRed to reveal neuronal architecture. Representative images of fixed neurons are displayed along with graph showing quantification of relative axonal EGFP-p35 levels. Scale bar = 20 μm. Data were analysed by Welch’s ANOVA and Games-Howell post hoc test; *N* = 30–34, error bars are s.e.m., *p < 0.05, **p < 0.01. **b** Representative kymographs showing axonal transport of DsRed-p35 in living neurons; for clarity anterogradely moving DsRed-p35 are illustrated alongside, time = 180 s. Graph shows quantification of anterogradely moving DsRed-p35 in the different treated neurons. Data were analysed by Welch’s ANOVA and Games-Howell post hoc test; *N* = 17–26, error bars are s.e.m., *p < 0.05

The above findings suggest that LMTK2 may function to facilitate anterograde axonal transport of p35 on kinesin-1-KLC1 motors. We therefore monitored axonal localization of transfected EGFP-p35 in untreated rat cortical neurons or in neurons treated with control or LMTK2 siRNAs. p35 was detected via its EGFP-tag and neuronal morphology determined by co-transfection with Ds-Red as described above. In agreement with previous studies, EGFP-p35 was present in cell bodies and axons in control neurons [18, 20, 36]. However, siRNA loss of LMTK2 markedly reduced EGFP-p35 axonal levels. To quantify this reduction, we normalized axonal fluorescent EGFP-p35 signals to those in cell bodies in a manner similar to that described above for quantifying axonal localization of LMTK2. These studies revealed a significant reduction in axonal EGFP-p35 in LMTK2 siRNA knockdown neurons (Fig. 5a).

To complement these studies, we also monitored whether siRNA loss of LMTK2 affected axonal transport of transfected DsRed-p35 in living rat cortical neurons using time-lapse microscopy. As was the case for EGFP-LMTK2, movement of DsRed-p35 was observed in both anterograde and retrograde directions with a mean anterograde velocity of 0.62 +/− 0.46 μm/s. This velocity was not significantly different to that observed for EGFP-LMTK2 (see above) and is again similar to those reported for other known kinesin-1/KLC1 cargoes such as calsyntenin-1, APP and Kidins220/ARMS [4, 50, 51]. However, compared to untreated or control siRNA treated neurons, siRNA loss of LMTK2 induced a significant reduction in anterograde movement of EGFP-p35 (Fig. 5b). Together, these findings together demonstrate that LMTK2 is required for proper axonal delivery and anterograde axonal transport of p35.

We also calculated how siRNA loss of LMTK2 affected the proportion of total moving p35 cargoes (anterograde plus retrograde) and their anterograde velocities. Compared to control siRNA, LMTK2 siRNA treatment did not significantly affect either of these parameters although there was a trend for a reduction in total movement. It is possible that increasing sample size to generate greater statistical power will reveal a significant affect.

### siRNA loss of LMTK2 also disrupts axonal transport of cdk5

**Fig. 6:**
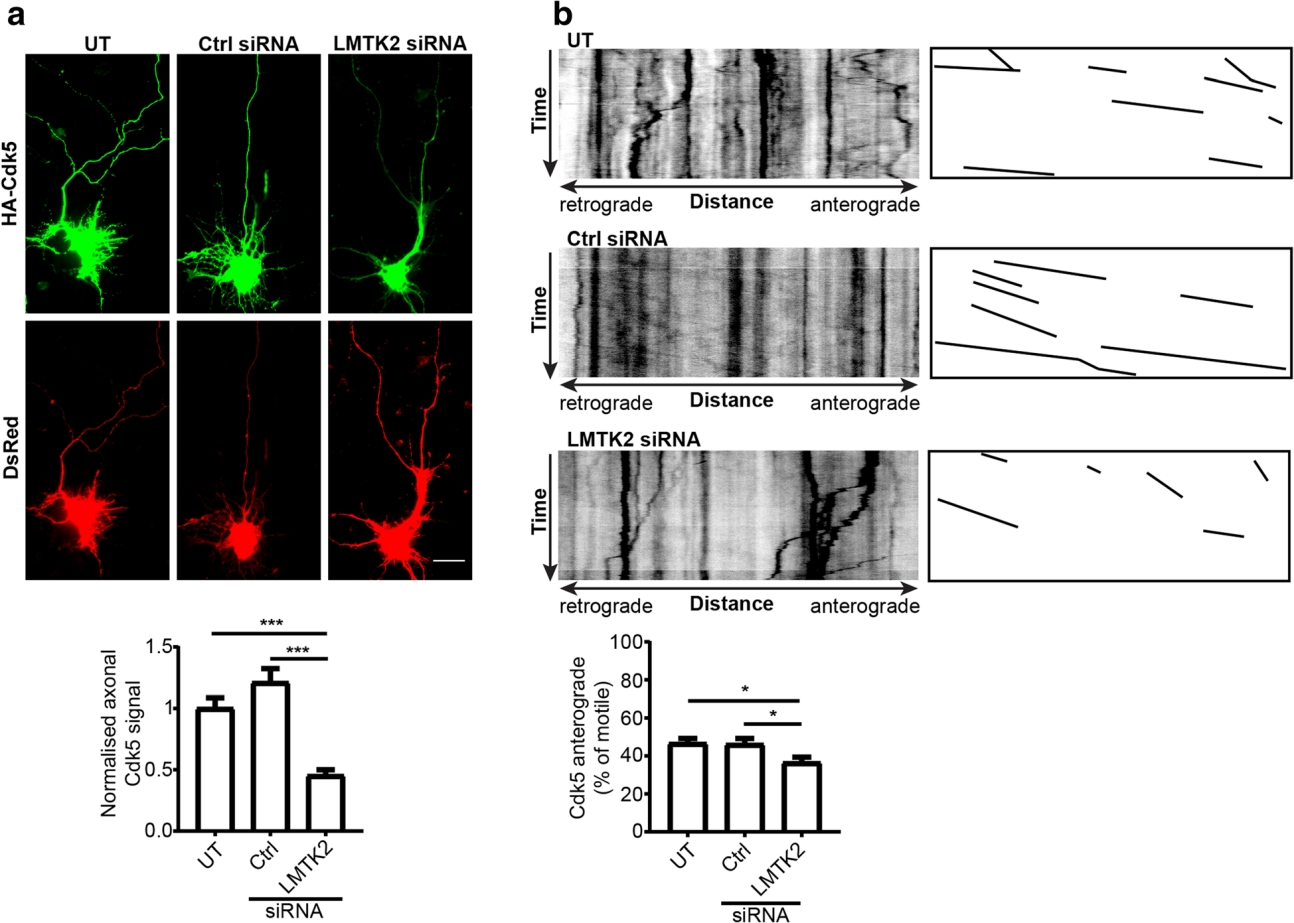
siRNA loss of LMTK2 reduces axonal levels and anterograde axonal transport of cdk5. **a** Neurons were either untreated (UT) or treated with control or LMTK2 siRNAs and then co-transfected with HA-cdk5 and DsRed to reveal neuronal architecture. Representative images of fixed neurons are displayed along with graph showing quantification of relative axonal HA-cdk5 levels detected using the epitope tag. Scale bar = 20 μm. Data were analysed by Welch’s ANOVA and Games-Howell post hoc test; *N* = 41–43, error bars are s.e.m., ***p < 0.001. **b** Representative kymographs showing axonal transport of EGFP-cdk5 in living neurons; for clarity anterogradely moving EGFP-cdk5 are illustrated alongside, time = 180 s. Graph shows quantification of anterogradely moving EGFP-cdk5 in the different treated neurons. Data were analysed by Welch’s ANOVA and Games-Howell post hoc test; *N* = 14–21, error bars are s.e.m., *p < 0.05

We also enquired whether siRNA loss of LMTK2 might disrupt axonal transport of cdk5 in a manner similar to that of its binding partner p35. To do so, we first monitored axonal localization of transfected HA-cdk5 in untreated rat cortical neurons or in neurons treated with control or LMTK2 siRNAs. Transfected cdk5 was detected by immunostaining for the HA-tag and neuronal morphology determined by co-transfection with Ds-Red as described above. In agreement with previous studies, HA-cdk5 was present in cell bodies and axons in control neurons [20, 36]. However, siRNA loss of LMTK2 markedly reduced axonal levels of HA-cdk5. To quantify this reduction, we normalized axonal fluorescent HA-cdk5 signals to those in cell bodies in a manner similar to that described above for quantifying axonal localization of LMTK2 and p35. These studies revealed a significant reduction in axonal HA-cdk5 in LMTK2 siRNA knockdown neurons (Fig. 6a).

We also enquired whether siRNA loss of LMTK2 affected axonal transport of transfected EGFP-cdk5 in living rat cortical neurons using time-lapse microscopy. As was the case for EGFP-LMTK2 and DsRed-p35, movement of EGFP-cdk5 was observed in both anterograde and retrograde directions with a mean anterograde velocity of 0.57 +/− 0.4 μm/s. This velocity was not significantly different to those we observed for EGFP-LMTK2 or DsRed-p35 (see above). However, compared to untreated or control siRNA treated neurons, siRNA loss of LMTK2 induced a significant reduction in anterograde movement of EGFP-p35 (Fig. 5b). Together, these findings demonstrate that LMTK2 is also required for proper axonal delivery and anterograde axonal transport of cdk5.

Finally, we calculated how siRNA loss of LMTK2 affected the proportion of total moving cdk5 cargoes (anterograde plus retrograde) and their anterograde velocities. Compared to control siRNA, LMTK2 siRNA treatment significantly reduced both of these features (total movement reduction *p* ≤ 0.001; velocity reduction *p* ≤ 0.001). Since LMTK2 siRNA loss had only minor effects on total movement of p35 (see above), it is possible that these more potent effects on cdk5 reflect the need for additional protein-protein interactions for cdk5 transport. Thus, whilst p35 transport involves formation of a tripartite KLC1-LMTK2-p35 complex, cdk5 transport requires a quaternary complex of KLC1-LMTK2-p35-cdk5.

### LMTK2 levels are reduced in cortex but not cerebellum of Alzheimer’s disease patients

**Fig. 7:**
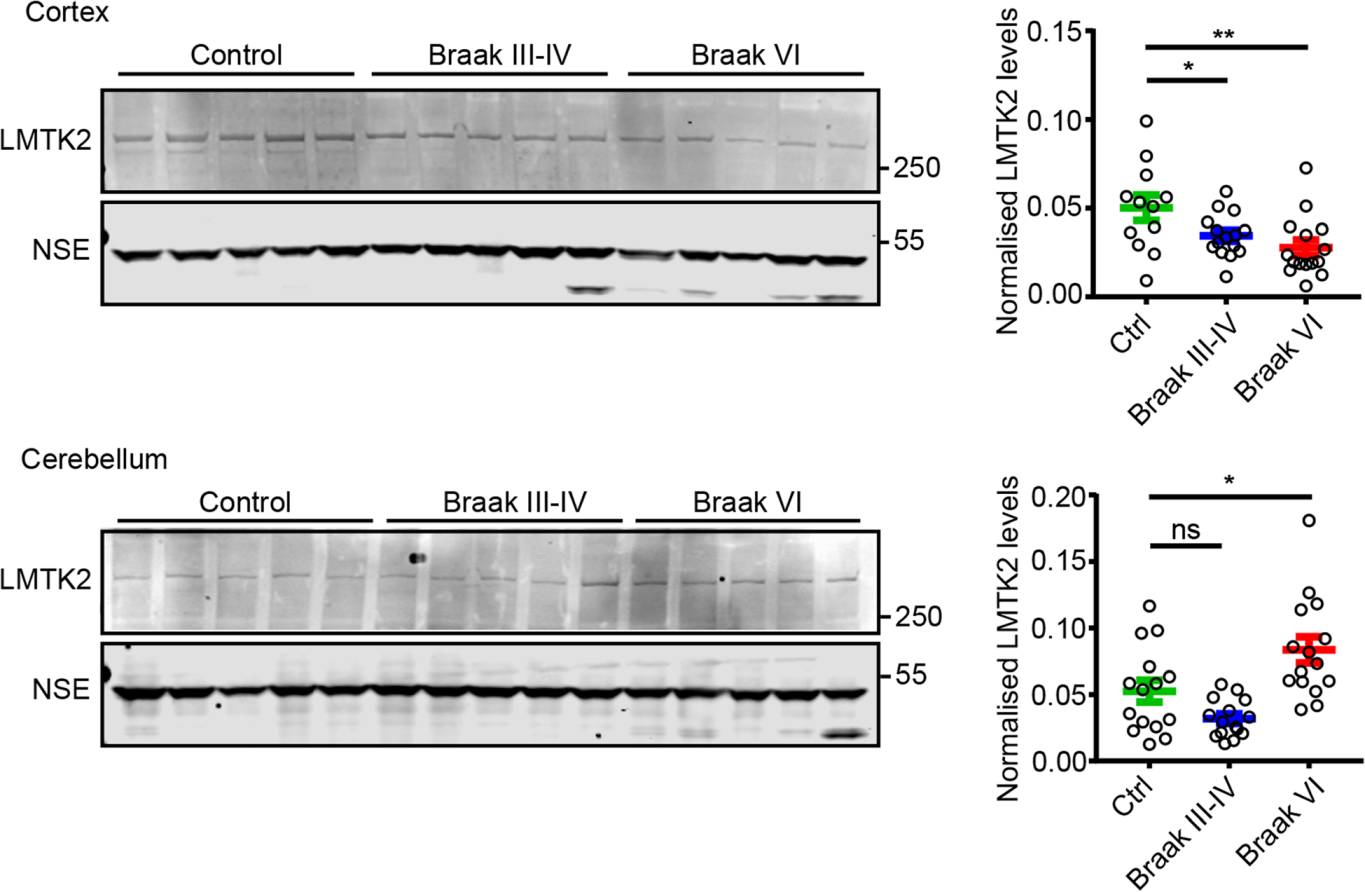
LMTK2 levels are reduced in post mortem Alzheimer’s disease cortex but not cerebellum. Representative immunoblots showing LMTK2 and NSE levels in post mortem human control (Ctrl) and Alzheimer’s disease cortex and cerebellum. Graphs show quantification of LMTK2 levels in the different samples normalised to NSE levels. Data were analysed by one-way ANOVA and Holm-Sidak post hoc test, error bars are s.e.m., *p < 0.05 **p < 0.01, ns not significant

Disruption to axonal transport is a major pathogenic event in Alzheimer’s disease [2, 5, 9, 30, 55]. Since siRNA loss of LMTK2 perturbed axonal transport of both p35 and cdk5, we enquired whether LMTK2 levels might be altered in Alzheimer’s disease. To do so, we monitored LMTK2 levels by immunoblotting in post-mortem Alzheimer’s disease brain tissues. Details of these human samples are shown in Table 1; there were no significant differences in age or post-mortem delay between the Alzheimer’s disease and control cases. We studied LMTK2 levels in cortex and cerebellum (affected and non-affected regions) in control, Braak stage III-IV (mid dementia) and Braak stage VI (severe dementia) cases. We normalised LMTK2 levels to the levels of NSE as described by others [21, 45]. Compared to controls, LMTK2 levels were reduced in cortex in both Braak stage III-IV and stage VI cases. By contrast, in cerebellum LMTK2 levels were unaffected in Braak stage III-IV but were elevated in Braak stage VI cases (Fig. 7). These data suggest that loss of LMTK2 in dementia may contribute to neuronal dysfunction by disruption of axonal transport of key cargoes through axons.

**Table 1:** Data for human post-mortem samples showing age at death, sex, post-mortem delay and pathological diagnosis

Case Group	Autopsy Code	Sex	Age	Post-Mortem Delay (hrs)	Braak stage
Control	A002/13	M	90	45	–
Control	A007/15	F	74	64	II
Control	A033/11	M	82	47	I
Control	A046/12	F	92	9	II
Control	A048/09	M	82	18	–
Control	A063/10	F	90	50	II
Control	A082/14	M	68	60	II
Control	A105/14	F	77	21	–
Control	A158/14	F	73	27	I
Control	A209/13	M	80	55	II-III
Control	A213/12	M	78	24	III
Control	A308/09	M	66	52	–
Control	A319/14	F	90	44	II
Control	A346/10	F	84	34	I-II
Control	A407/13	F	80	22	II
AD Braak III-IV	A037/04	F	96	39	IV
AD Braak III-IV	A041/04	F	97	67.5	III-IV
AD Braak III-IV	A065/16	M	91	48	IV
A DBraak III-IV	A067/09	F	92	19.5	III
AD Braak III-IV	A078/13	M	86	52.5	IV
AD Braak III-IV	A084/16	F	86	55.5	IV
AD Braak III-IV	A097/13	M	82	28	IV
AD Braak III-IV	A101/08	F	92	29.5	IV
AD Braak III-IV	A189/07	F	83	41.5	IV
AD Braak III-IV	A223/12	F	83	22	IV
AD Braak III-IV	A232/16	F	95	47	IV
AD Braak III-IV	A282/11	M	93	13.5	IV
AD Braak III-IV	A374/14	M	88	79	III-IV
AD Braak III-IV	A378/14	M	98	53	IV
AD Braak III-IV	A381/16	M	84	86	IV
AD Braak VI	A008/12	M	66	41	VI
AD Braak VI	A064/16	F	93	49	VI
AD Braak VI	A087/16	F	89	38.5	VI
AD Braak VI	A100/15	F	73	30	VI
AD Braak VI	A105/13	F	81	17.5	VI
AD Braak VI	A166/12	F	86	25	VI
AD Braak VI	A171/14	M	84	67	VI
AD Braak VI	A226/16	F	69	73	VI
AD Braak VI	A258/16	M	67	39.5	VI
AD Braak VI	A289/13	M	83	22	VI
AD Braak VI	A331/15	M	86	38	VI
AD Braak VI	A342/14	F	84	27	VI
AD Braak VI	A355/14	F	79	31	VI
AD Braak VI	A377/14	F	85	79	VI
AD Braak VI	A380/13	F	81	20	VI

*AD* Alzheimer’s disease, *F* female, *M* male

## Discussion

Here we show that LMTK2 binds to kinesin-1 light chains and that this involves interactions between the KLC TPR domains and a C-terminal WD motif (WD1388/1389) in LMTK2. We also demonstrate that binding of LMTK2 to KLCs facilitates its transport into and through axons of neurons. Thus, mutation of LMTK2(WD1388/1389) to inhibit binding to KLCs and siRNA loss of KLC1 both reduce axonal LMTK2 levels and anterograde axonal transport of LMTK2 in living neurons.

LMTK2 also binds to the cdk5 activator protein p35 [18]. cdk5/p35 is a key neuronal kinase that regulates a number of fundamental cellular functions in both axons and synapses but the mechanisms by which it is transported to these locations are unknown [20, 43]. We show that LMTK2 mediates the formation of a KLC1-LMTK2-p35 complex and that siRNA loss of LMTK2 perturbs axonal transport and axonal delivery of p35. We also show that siRNA loss of LMTK2 disrupts axonal transport of cdk5. Together, these findings demonstrate that LMTK2 is required for proper axonal delivery and anterograde axonal transport of both p35 and cdk5.

siRNA knockdown of KLC1 and mutation of LMTK2(WD1388/1389) both reduced but did not eliminate axonal LMTK2 levels and anterograde axonal transport of LMTK2. The remaining transport of LMTK2 in the KLC1 siRNA knockdown neurons may be due to incomplete siRNA loss of KLC1 or the presence of KLC2 which we show also binds to LMTK2. Likewise, the remaining anterograde movement of mutant LMTK2(WD1388/1389AA) may be due to movement on KLC2 containing motors since we show that the LMTK2 WD mutations abrogate binding to KLC1 but only reduce binding to KLC2. Others have described differences in binding of WD/WE containing cargo proteins to KLC1 and KLC2 [10, 49].

In a similar fashion, knockdown of LMTK2 did not completely block transport of p35 and cdk5. This could again be a consequence of incomplete siRNA loss of LMTK2. Interestingly however, a further member of the LMTK family, LMTK1 (also known as AATYK1) has also been shown to bind to p35 so it is possible that LMTK1 acts to scaffold p35 to KLCs in a manner similar to that of LMTK2 [48]. Indeed, like LMTK2, LMTK1 contains a conserved C-terminal WD motif. Thus, at least two members of the LMTK family may function as scaffolding ligands for KLCs.

Damage to p35 and disruption of cdk5/p35 activity are strongly linked to Alzheimer’s disease [7, 41, 43]. Moreover, abnormal cell body accumulation of cdk5/p35 is observed in affected regions in post-mortem Alzheimer’s disease brains [25, 38, 39, 57]. Such accumulations are consistent with disruption to axonal transport of cdk5/p35. Damage to axonal transport is a major feature of Alzheimer’s disease [2, 5, 9, 30, 55]. Of interest therefore were our findings that LMTK2 levels are reduced in Alzheimer’s disease cortex (affected region) and that this reduction occurs in mid (Braak stage III-IV) as well as severe (Braak stage VI) dementia cases. This loss of LMTK2 in Braak stage III-IV cortex suggests that it is may be an early pathogenic feature; early pathogenic events are generally believed to contribute most to the disease process. In contrast to the Alzheimer’s disease cortex, LMTK2 levels were elevated in Braak stage VI cerebellum. Whether this increase in cerebellar LMTK2 levels contributes to any protection from the neurodegenerative process in this brain region is not clear. Since we show that LMTK2 mediates axonal transport of p35 and cdk5, this loss of LMTK2 may contribute to the cell body cdk5/p35 accumulation that has been described in Alzheimer’s disease [25, 38, 39, 57].

Aside from binding to KLCs and p35, LMTK2 also interacts with the molecular motor protein myosin VI and the catalytic subunit of protein phosphatase-1 (PP1C) [8, 16, 27, 53]. These interactions involve domains within LMTK2 that are distinct from the KLC WD interaction motif that we identify here. The LMTK2-myosin VI interaction regulates endosome trafficking and delivery of proteins to axonal membranes [8, 16, 24]. The interactions between cdk5/p35, LMTK2 and PP1 have been shown to form a signaling pathway that regulates the activity of glycogen synthase kinase-3β (GSK-3β) [27, 28, 32]. Like cdk5/p35, PP1C and GSK-3β are key signaling molecules within neurons [15, 35]. In this pathway, cdk5/p35 phosphorylates and activates LMTK2 which in turn induces inhibitory phosphorylation of PP1C on threonine-320; this leads to increased inhibitory phosphorylation of GSK-3β on serine-9. One target of this signaling involves GSK-3β phosphorylation of KLC2 which causes release of some cargoes to inhibit their transport [27, 32].

Thus, LMTK2 is centrally placed to regulate a number of fundamental transport processes within axons including those driven by kinesin-1 and myosin VI. This regulation may be via scaffolding functions that deliver LMTK2 and interacting partners such as p35 and cdk5 to axons as we describe here, and also signaling functions involving cdk5/p35, PP1C and GSK-3β activities. Moreover, like cdk5/p35, both PP1 and GSK-3β are strongly linked to the pathogenesis of Alzheimer’s disease [6, 14, 22, 26]. The loss of LMTK2 that we describe in Alzheimer’s disease brains may therefore impact on both axonal transport and neuronal signaling functions which are all perturbed in dementia. Our discovery that LMTK2 is a KLC binding protein that facilitates axonal transport of cdk5/p35 provide the basis for a proper dissection of such functions. They also pave the way for further studies of LMTK2 in Alzheimer’s disease post-mortem tissues and in transgenic mouse models of Alzheimer’s disease. In this context it is noteworthy that as we show in the post-mortem human tissues, LMTK2 levels are also reduced in cortex and increased in cerebellum in some transgenic mouse models of Alzheimer’s disease [29]. Future studies could therefore include correlating levels of LMTK2 to those of phosphorylated Tau, Aβ, cdk5, p35 and GSK3β, and complementary immunohistochemical studies of LMTK2, Tau, cdk5 and p35.

## Conclusions

LMTK2 is a neuronal serine/threonine kinase that binds to the cdk5 activator subunit p35. Here we show that LMTK2 also binds to KLC1/2 and this facilitates its transport into and through axons. The interaction involves the TPR domains in KLC1/2 and a WD motif in the C-terminus of LMTK2. We also show that LMTK2 facilitates the formation of a complex containing KLC1 and p35 and that siRNA loss of LMTK2 disrupts axonal transport of both p35 and cdk5. Finally, we show that LMTK2 levels are reduced in affected cortical regions in post-mortem Alzheimer’s disease brains. Defective cdk5/p35 activity and damage to axonal transport are both features of Alzheimer’s disease. Thus, LMTK2 binds to KLC1 to direct axonal transport of p35 and its loss may contribute to Alzheimer’s disease.

## Additional file

Additional file 1: **Figure S1.** siRNA knockdown of KLC1 and LMTK2 in rat cortical neurons. Knockdown of KLC1 does not affect expression of LMTK2, kinesin-1, KLC1, p35, cdk5 or tubulin. siRNA knockdown of LMTK2 in rat cortical neurons does not affect expression of kinesin-1, KLC1, p35, cdk5 or tubulin. siRNAs for LMTK2 and KLC1 have been described previously [27, 51]. **Figure S2.** Characterisation of KLC1-p35 PLAs in rat primary neurons. PLAs were performed with no primary antibodies, goat anti-KLC1 antibody alone, rabbit anti-p35 antibody alone or both KLC1 and p35 antibodies. Scale bar = 20 μm. Graph shows quantification of PLA signals in the different treated neurons. Neurons we also immunostained for tubulin to reveal neuronal architecture. Arrows show KLC1-p35 signals in axons. Data were analysed by Welch’s ANOVA and Games-Howell post hoc test; *N* = 25, ***p < 0.001. (DOCX 225 kb)

## Abbreviations

AA: Amino acid
ANOVA: Analysis of variance
APP: Amyloid precursor protein
ATP: Adenosine triphosphate
Cdk5: Cyclin dependent kinase-5
EGFP: Enhanced green fluorescent protein
GSK-3β: Glycogen synthase kinase-3β
HA: Hemagglutinin
HRP: Horseradish peroxidase
IB: Immunoblot
IF: Immunofluorescence
IP: Immunoprecipitation
KLC: Kinesin-1 light chains
LMTK2: Lemur tyrosine kinase-2
NSE: Neuron specific enolase
PBS: Phosphate buffered saline
PLA: Proximity ligation assay
RIPA: Radioimmunoprecipitation assay
SDS-PAGE: Sodium dodecyl sulphate polyacrylamide gel electrophoresis
SEM: Standard error of mean
siRNA: Small interfering RNA
TBS: Tris-HCl buffered saline
TPR: Tetratricopeptide repeat
