# Supplementary material for: LMTK2 binds to kinesin light chains to mediate anterograde axonal transport of cdk5/p35 and LMTK2 levels are reduced in Alzheimer’s disease brains
Source: Acta Neuropathol Commun. 2019 May 8;7:73. doi: 10.1186/s40478-019-0715-5 (PMC6505310; doi:10.1186/s40478-019-0715-5)
Supplement: Supplementary file 1 — Figure S1. siRNA knockdown of KLC1 and LMTK2 in rat cortical neurons. Knockdown of KLC1 does not affect expression of LMTK2, kinesin-1, KLC1, p35, cdk5 or tubulin. siRNA knockdown of LMTK2 in rat cortical neurons does not affect expression of kinesin-1, KLC1, p35, cdk5 or tubulin. siRNAs for LMTK2 and KLC1 have been described previously [27, 51]. Figure S2. Characterisation of KLC1-p35 PLAs in rat primary neurons. PLAs were performed with no primary antibodies, goat anti-KLC1 antibody alone, rabbit anti-p35 antibody alone or both KLC1 and p35 antibodies. Scale bar = 20 μm. Graph shows quantification of PLA signals in the different treated neurons. Neurons we also immunostained for tubulin to reveal neuronal architecture. Arrows show KLC1-p35 signals in axons. Data were analysed by Welch’s ANOVA and Games-Howell post hoc test; N = 25, ***p < 0.001. (DOCX 225 kb) [file 40478_2019_715_MOESM1_ESM.docx]

**Additional file 1**


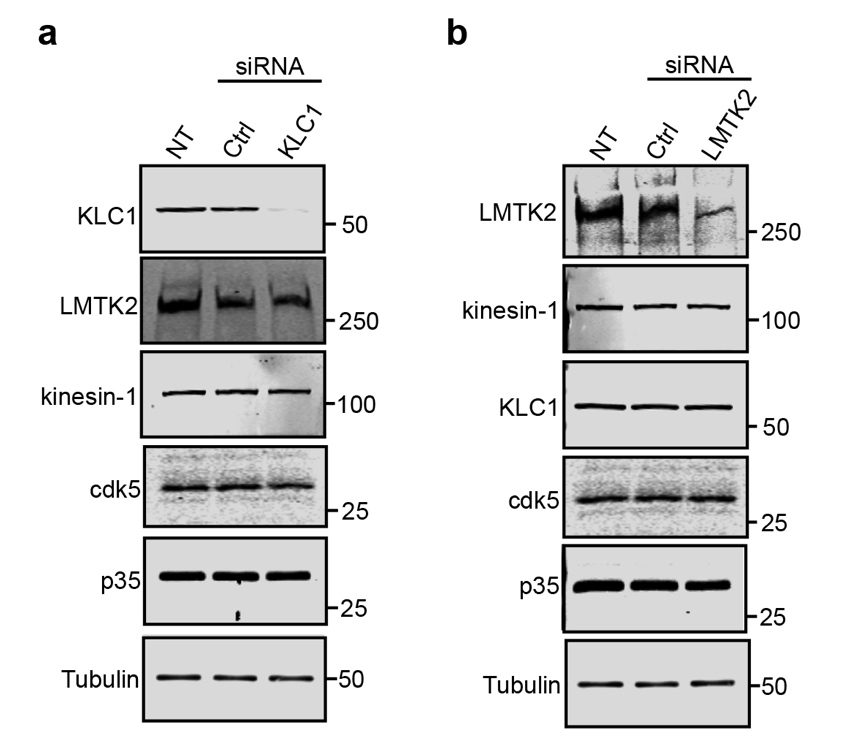


Figure S1. siRNA knockdown of KLC1 and LMTK2 in rat cortical neurons. Knockdown of KLC1 does not affect expression of LMTK2, kinesin-1, KLC1, p35, cdk5 or tubulin. siRNA knockdown of LMTK2 in rat cortical neurons does not affect expression of kinesin-1, KLC1, p35, cdk5 or tubulin. siRNAs for LMTK2 and KLC1 have been described previously [27, 51].


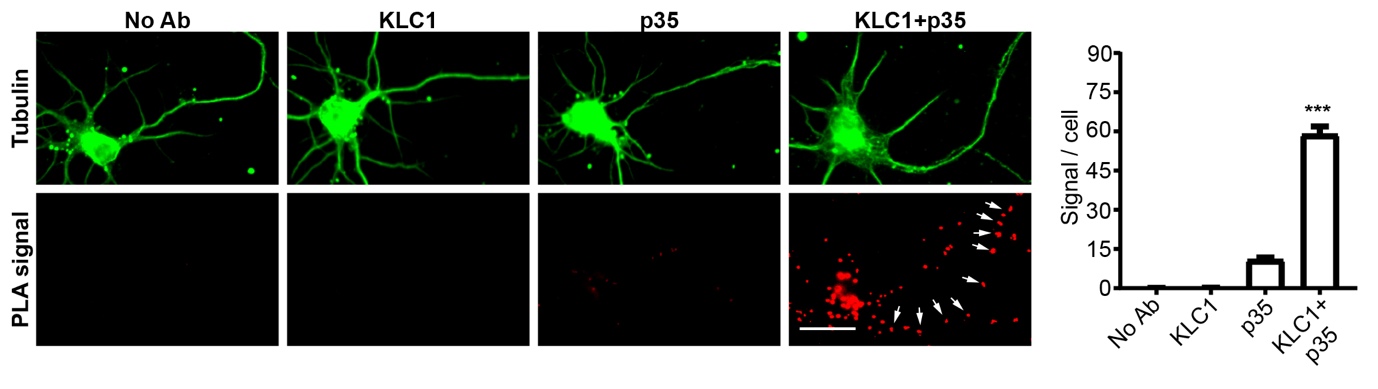


Figure S2. Characterisation of KLC1-p35 PLAs in rat primary neurons. PLAs were performed with no primary antibodies, goat anti-KLC1 antibody alone, rabbit anti-p35 antibody alone or both KLC1 and p35 antibodies. Scale bar=20 μm. Graph shows quantification of PLA signals in the different treated neurons. Neurons we also immunostained for tubulin to reveal neuronal architecture. Arrows show KLC1-p35 signals in axons. Data were analysed by Welch’s ANOVA and Games-Howell post hoc test; N=25, ***p<0.001.
